# The domestication of Foucault: Government, critique, war

**DOI:** 10.1177/0952695114538990

**Published:** 2014-12

**Authors:** Ansgar Allen, Roy Goddard

**Affiliations:** University of Sheffield, UK; University of Sheffield, UK

**Keywords:** critique, Michel Foucault, governmentality, power, revolution

## Abstract

Though Foucault was intrigued by the possibilities of radical social transformation, he resolutely resisted the idea that such transformation could escape the effects of power and expressed caution when it came to the question of revolution. In this article we argue that in one particularly influential line of development of Foucault’s work his exemplary caution has been exaggerated in a way that weakens the political aspirations of post-Foucaldian scholarship. The site of this reduction is a complex debate over the role of normativity in Foucaldian research, where it has been claimed that Foucault’s genealogical approach is unable to answer the question ‘*Why fight?*’ The terms of this debate (on the neo-Foucaldian side) are limited by a dominant though selective interpretation of Foucault’s analytics of power, where power is understood primarily in terms of government, rather than struggle. In response we suggest that if we reconfigure power-as-government to power-as-war, this adjusts the central concern. ‘*Why fight?*’ becomes replaced by the more immediate question, ‘*How fight?*’ Without denying the obvious benefits of cautious scholarly work, we argue that a reconfiguration of Foucault’s analytics of power might help Foucaldian research to transcend the self-imposed ethic of political quietism that currently dominates the field.

## Introduction

We reproach this world not for going to war too ferociously, nor for trying to prevent it by all means;we only reproach it for reducing war *to its most empty and worthless forms.* (Tiqqun, 2010: 59)

Cigar in hand Jacques Lacan looks on as a young man pours water over his desk. It is 13 October 1972. Outwardly calm, Lacan encourages the man to explain what he hoped to achieve by interrupting the lecture. ‘To make revolution’ is the reply. This moment, he declares, has been chosen to denounce intellectuals who preach its impossibility, providing a rationale for their own political acquiescence. The interruption is instructive on two counts: first, it dramatizes a difference in comportment between the would-be revolutionary who acts, and the intellectual who stands by to observe, transforming moments of action to points of reflection; second, the heckler takes issue with the potential *effects* of Lacan’s ideas rather than with their internal consistency. This article adopts a similar strategy, which it applies to the work and legacy of a different thinker. It is an investigation into the political effects of Michel Foucault’s work. As is well known, Foucault, like the young man in question, had his own critique of the intellectual. Foucault’s critique is similar in that it, too, takes issue with the problem of intellectual detachment (Foucault, 1977a). We claim, nevertheless, that despite Foucault’s best efforts, the young revolutionary might well judge Foucault or at least his present-day followers to be deserving of the same watery treatment.

## The critical challenge

We claim that in the political climate of our present, Foucault’s ideas have been largely emptied of their political force. In part, this is because they were designed to respond to the pressures of a very different context. When Foucault (1998[1976a]: 82) established his ‘analytics of power’ in the 1970s he was responding to the critical challenges of that decade. Take, for example, his discussion with Maoists on the topic of popular justice (Foucault, 1980a[1972a]). In this discussion, Foucault’s analytic caution comes across as an essential counter to militant presumption, and yet Foucault remains open to the possibility of revolutionary change. For instance, while his interlocutor speculates that the liquidation of bosses in a country the size of France might not be practical, and that popular justice requires the careful guidance of a revolutionary court, Foucault patiently explains that the Maoists are already assuming too much. Their problem is more fundamental. The structure of a court, the idea of a neutral third party, the processes of power and enforcement, and the reference to a ‘universal’ idea of justice, are all embedded in the old order. In making uncritical use of such devices, the revolutionary state apparatus will unwittingly extend and support elements of the regime that it sought to overthrow. To prevent such mistakes, revolutionary terror, if it can be supported at all, requires *better calibration*.

To speak of ‘calibrating terror’ looks odd almost half a century later, if not offensive to good taste. When it comes to revolution, the bitter flavours left by a century of political experimentation have bred a delicate appetite. As Slavoj Žižek (2007a: viii) argues when speaking about the French Revolution: almost everyone, ‘today’s “radical Left” included, is now somehow ashamed of the Jacobin legacy of revolutionary terror with its state-centralized character’. Those who seek to reform the left so that it may regain some measure of political effectiveness tend to favour ‘free interaction’ and ‘multiple subjectivities’ against the ‘centralized hierarchy’ and singular Truth of a Jacobin-styled revolution (ibid.). Pluralized, piecemeal and peaceable, these are the only methods of refusal conceivable today, unless, that is, you live outside the liberal-democratic West.^1^ Back home, anything radical, disruptive, or potentially violent is to be deplored. For Žižek this disposition means that the contemporary left concedes its own defeat before it has got its boots on.

To succeed, a revolution must be fundamental in its reach, so the argument goes. In what appears to be a deliberate attempt to upset liberal-left sensibilities Žižek (2007b: 195) claims that revolutions have failed hitherto not because they were ‘too extreme’ but because they ‘did not question their own presuppositions’ adequately enough. As we have seen, Foucault (2002a[1977b]: 123) would presumably agree here with Žižek, having argued himself that one can ‘perfectly well conceive of revolutions that leave essentially untouched the power relations that form the basis of the functioning of the state’. Indeed, communist regimes may well have ‘reinvested the very power-mechanisms constituted by the capitalist State’. In part, for Foucault, this was because ‘the problem of the mechanics of power or the mechanisms of power [was not adequately] posed or analysed’ (Foucault, 2004[1976b]: 261). Radical, emancipatory theory failed to anticipate these reactionary outcomes because of its tendency to reduce the complexities of power to simplistic relations of domination and exploitation.

It has been claimed that Foucault’s position with regard to revolution was so exacting that it announced (and even affirmed) the disappearance of revolution and the onset of a ‘post-revolutionary’ age. This is the view, indeed, of Foucault’s former student, collaborator and de facto executor of his intellectual estate, François Ewald. In the 1970s, so the story goes, Foucault correctly identified a change in the consciousness of time. This transformation took us beyond history, to an ‘end of history’ in which time was converted ‘into a sort of infinite space’, becoming an ‘eternal present’ where one ‘just keeps oneself busy, because nothing more can happen’ (Ewald, 1999: 85–6). While Ewald’s description of our present is certainly recognizable, it is nevertheless problematic in Ewald’s hands. This diagnosis of his is matched to a personal career in which a former Maoist of the ’68 generation has come to terms with failure by abandoning all revolutionary hope and explaining its impossibility through an ‘antirevolutionary’ interpretation of Foucault’s work (see Behrent, 2010).

While our ‘eternal present’ does indeed put forward a distinct challenge to revolutionary action, we claim that Foucault provides some of the tools required to tackle it. Admittedly, these tools were developed in opposition to the polarizing and reductive effects of revolutionary discourse as it appeared to Foucault in the early 1970s. However, these tools were also designed *to supersede* this problematic discourse. Foucault challenged the Marxist tendency to overly reductive denunciations of power, not simply in order to sensitize political analysis, but also to *broaden the scope of political struggle*. Recovering ex-Marxists such as Ewald tend to emphasize the opportunities Foucault afforded to political analysis rather than those he afforded to struggle.

As is well known, Foucault (2000a[1971a]: 369) recommended a profusion of ‘grey, meticulous, and patiently documentary’ investigations. These investigations would *not* consider society as a whole and then recommend universal solutions, for ‘“the whole of society” is precisely that which should not be considered except as something to be destroyed’ (Foucault, 1980b[1971b]: 233). In other words, speaking at this level of abstraction generates a form of analytic blindness that allows old forms of power to be smuggled through, including the tyrannical notion that society can be ordered according to a single unifying law. Since power is radically dispersed and locally contingent its overthrow ‘does not, then, obey the law of all or nothing’. Power is ‘not acquired once and for all by a new control of the apparatuses nor by a new functioning or a destruction of the institutions’ (Foucault, 1991[1975a]: 27). The revolutionary challenge is to unveil power and overthrow it in all its dispersion and specificity.

The analytic consequences of this position can be summarized like this: (1) when power is seen as widely dispersed rather than located in one particularly powerful and coercive institution, this diminishes the importance of the question of legitimacy in analyses of power since no one agent or group of agents can be held accountable; (2) once power is believed to be operating throughout society rather than emanating from the centre, this acts to disarm any theory of politics and power based on opposition to the state; (3) although this approach bears some similarities to critical theory, according to which ‘instrumental rationality’ has spread throughout western societies killing off ethical rationalities, Foucault does not see a single, uniform strain of this rationality. Instead, he identifies a range of local and contingent rationalities. This theoretical switch changes the perceived role of intellectual or critic. Without reference to a dominant principle or underlying rationale, critique can no longer adopt the ‘premise of a deduction that concludes, “this, then, is what needs to be done”’ (Foucault, 2002b[1978a]: 236). To make such recommendations would demand precisely the sort of global and unitary view that is no longer deemed possible.

Foucault did, nevertheless, successfully maintain a critical ethos that was able to operate outside of conventional politics. This ethos was combined with a definite refusal to translate his work into the terms of political debate:

The Communist or Socialist Parties have never put on their working agenda the analysis of the power of reason over unreason [which was Foucault’s early research agenda]. Perhaps that is not their job. But if it isn’t their problem, theirs is not necessarily mine either. (Foucault, 2002c[1978b]: 286; parenthesis added)

As a critical intellectual with a growing reputation, Foucault was desperate to avoid becoming the ‘alter ego, double, or alibi’ of any existing party or social movement (2002c[1978b] 288). Here one can measure the success of Foucault’s work by the distance it maintains not only from mainstream politics, but also from the conventions of a more radical politics, from the received wisdom of those seeking to overthrow political systems according to theories of society that Foucault would argue are embedded in and thus bound up with the systems they seek to replace.

Aiming to operate without the security of foundational claims Foucault developed a mode of enquiry to match his ethos. As Thomas Lemke (2011: 29) observes, critique is usually limited to a practice ‘characterized by deficit, dependency and distance’. (1) It perceives ‘epistemological problems’ as cognitive errors requiring correction – it corrects deficits in understanding. (2) It is dependent on something other than itself, a ‘normative infrastructure that specifies its lawful foundation and its legitimate objective’. (3) It establishes a system of distance, creating an opposition between ‘those who know and those who do not’ (ibid.: 29–30).

As we explain below, Foucault transformed the critical enterprise by (1) exploring the frameworks imposed on the production of knowledge and the operations of power; (2) relating each normative infrastructure to its historical genesis in regimes of knowledge and power; and (3) working within these normative frameworks in order to render them unstable, liable to collapse and *open to reconstruction* on different terms by those who were once subjected to them.

If we are to believe Foucault’s occasional and more obviously militant remarks, this determination to localize and particularize problems, to eschew legislative statements and political allegiances, to avoid in other words the usual tendencies of critique, does not reduce the critical enterprise, as if by default, to a weakened state. Foucault speaks of his desire to ‘break through that rigid yet fragmented crust’ formed by the dogmas and ‘endless discussions’ of intellectual debate, and his desire for a ‘personal, physical, and real involvement’ in political events (Foucault, 2002c[1978b]: 281–2). Foucault was indeed no stranger to political protest, direct action, police brutality, arrest and deportation (see, for example, Macey, 1993: 205, 226, 270, 312, 341–52). With his own conduct in mind, Foucault argued that those who oppose the police must ‘not allow them the hypocrisy of masking physical force behind orders which have to be obeyed immediately’ (Foucault cited in ibid.: 350). He confessed admiration for practical revolutionaries, most famously commenting on the Iranian Revolution of 1978. This revolutionary moment was, in his view, praiseworthy for being relatively unencumbered by revolutionary expertise, by that ‘gigantic effort to domesticate revolts within a rational and controllable history’ (Foucault, 2002d[1979a]: 450). The Tunisian revolts of 1968 had a similar quality, in which, for Foucault, the ‘Marxist education of the Tunisian students was not very deep’ but their level of understanding ‘wasn’t essential’ (Foucault, 2002c[1978b]: 280). Theoretical ‘exactness’ or subtlety was not necessary for a revolt animated by desire, myth and spirituality, in which practical decisions were located in the ‘logic of events’ (Osborne, 1999: 51).

The point to note is that Foucault was not only prepared to engage directly with Marx (see Choat, 2010), he was also willing to engage with certain forms of Marxism. Here the crucial factor in Foucault’s appraisal of Marxist discourse was his estimation of the productivity of the relationship between Marxist theory and the accompanying logic of events. Foucault contrasts the ‘vehemence and intensity’ of appeals to Marxism in Tunisia, to the Marxism he experienced in France, in which the revolutionary impulse is reduced to the format of ‘cold academic discussion’. After May 1968 these discussions fragmented into a ‘proliferation of theories’, into an ‘irrepressible discursivity’ that was far removed, in his view, from the practical impulses of revolt (Foucault, 2002c[1978b]: 280–1). The practical-intellectual task, one might interpret, was to help shape an ‘enthusiasm for the Revolution’ (Foucault, 1983b: 18) by bringing into question all that appears ‘universal, necessary, obligatory’ (Foucault, 2000b[1984a]: 315) without then crushing such enthusiasm under the weight of cold academic discussion.

The challenges Foucault faced are, however, no longer our own. If Žižek is correct in suggesting that pluralized, piecemeal and peaceable political interventions are the only forms of revolt still acceptable for the majority on the left today, it becomes clear that the political context for critical theoretical work has radically altered. It could be argued that Foucault’s strategic position as it has been translated into the terms of neo-Foucaldian scholarship, finds itself complicit in this overall retreat of the left. This would be because it continues to adopt a fastidiously restrained scholarly stance designed for a radically different context; a context in which the organized left was still a force to be reckoned with. In mimicry of Foucault’s most analytically cautious tendencies, this scholarship has been prolific in its output, and yet, the weight of these enquiries is no longer placed against the forces of a widespread, organized militancy and its associated intellectual vanguard to which the neo-Foucaldian could respond with a derisive laugh. We are no longer faced with the theoretical globalisms of a widespread Marxism. Consequently any attempt to revive a project that replaces ‘universal’ with ‘specific intellectuals’ (Foucault, 2002a[1977b]: 127), for example, makes far less sense now that the left has been so successfully released from the intellectual grip of ‘universalist’ Marxism. The problem is that political problems are today almost entirely posed in specific, that is to say, immediate terms. Yet the most urgent political (and social and cultural) problems of the present are framed within a global context. Specificity alone fails to address the scale of the challenge presented.

## The Foucault effect

There is a notable tendency in the work of Foucault’s many followers to translate his analytic caution (which as we have seen was the product of a very different context) into a latter-day form of political quietism. This tendency is most obvious in the grouping that was announced by and through *The Foucault Effect* (Burchell, Gordon and Miller, 1991) – the influence of which was recently celebrated at ‘The Foucault Effect 1991–2011’ conference at Birkbeck College in London – and has been expressed most consistently but not exclusively through articles published in the journal *Economy and Society*, based at the London School of Economics. Prominent figures include Peter Miller, Nikolas Rose and Thomas Osborne. Indicative texts include those by Barry, Osborne and Rose (1996), Ashenden and Owen (1999), Dean (1999, 2007), Miller and Rose (2008), Rose (1999a, 1999b) and Rose, O’Malley and Valverde (2006). These authors wear their refusal to make normative – i.e. political – judgements as a badge of distinction. They celebrate the fact that they developed a mode of analysis that ‘*kept its distance* from the rhetorics of social critique’ (ibid.: 90; emphases added).

This tendency to translate Foucault’s analytic caution into political quiescence is not, in our view, limited to this largely British grouping. It can be observed in the work of many ‘western’ scholars across parts of continental Europe, Australia and North America, and is particularly prominent within traditions of neo-Foucaldian scholarship that work from the problematic of ‘governmentality’. Far more is at stake here than the quietism of a relatively small group of scholars working with Foucault and with the problematic of government. As these scholars themselves recognize, the ‘concepts and methodological choices utilized in governmentality studies’ spread so far, and with such notable success, precisely because they ‘resonated’ so well ‘with concurrent intellectual trends in a number of relatively independent fields’. This gave ‘the notion of governmentality and the research questions and perspectives associated with it traction across numerous disciplines, institutions, and geographical locations’ (Rose, O’Malley and Valverde, 2006: 92). It is worth asking, in response, what exactly it was that secured this rapid spread, and widespread adoption, causing such a wide range of institutions and disciplines to accept willingly these methodological (and for us also political) dogmas?

Our enquiry focuses on ‘the Foucault effect’ that has occurred within the intellectual contexts of those polities that define themselves as liberal democracies, which is to say, those societies in which radical left social analyses have been in decline. Elsewhere in, say, a post-colonial context such as Latin America, Foucault may have been taken up very differently (see, for example, Trigo, 2002). We argue that in the particular social and historical contexts of these ‘western’ democracies, a neo-Foucaldian ethic of research has come to dominate. In addition to the painstakingly descriptive and politically cautious approaches to researching power, government and subjectivity that characterize this tradition, some scholars within this loose grouping deliberately neglect their master theorist, as if undue reference to Foucault risks transforming a project that should meticulously uncover and describe the micro-operations of power into a project defined by the sort of theoretical conformity from which they are at pains to distance themselves. Nikolas Rose (1999a: ix) declares that it was a ‘deliberate decision’ to let his debt to Foucault remain ‘implicit’. We found how, when he was writing with Peter Miller, they ‘preferred not to be Foucault scholars’ (Miller and Rose, 2008: 8), advocating ‘a relation to his work that is looser, more inventive and more empirical’ (Rose, 1999b: 5). After all, was this not what Foucault sometimes did to Nietzsche and also to an extent Marx?^2^ He let them labour in the background and largely out of sight. The true Foucaldian, we are led to believe, does the same with Foucault.

When scholars reference Foucault more directly we encounter a recurring fear that they might nevertheless ‘pin Foucault down’ through an overly tidy explication of his thought (Huffer, 2010: 332). It is as if any strong statement about Foucault, or an aspect of his work, would automatically translate his ideas into a totalizing discourse. And so attempts are made to speak about Foucault ‘without ever producing a definitive object we call “Foucault”’ (ibid.). Known as the ‘author function’ (Foucault, 2000c[1969a]: 211) this type of reductive operation has been successfully avoided by many scholars. Those explications of Foucault’s *oeuvre* that do exist often begin with an apology, stating that overviews are impossible, particularly with Foucault, and that they risk exercising a restraint upon his work. While this determination to avoid pinning Foucault down makes intrinsic sense (where the intention is to encourage the proliferation of Foucault’s ideas rather than impose upon them the constraints of received interpretations of his work), this effort to keep Foucault open so as to maintain the versatility, productivity and indeed fecundity of his work, has become an dogma of its own. For example, the suggestion that Foucault had a militant streak that has been much neglected in recent work is swiftly rejected as the misguided project of those who would impose a biographical constraint upon his work. This is *not* our intention. We are concerned, nevertheless, by the effects of a dominant intellectual trend within Foucaldian scholarship that promotes an, as it were, undetermined, diffuse Foucault, an intellectual figure whose loosely assembled writing can be visited and savoured for its happily disengaged temper, and insists on a correspondingly unsystematized proliferation of his work and ideas. For us, this insistence that the convenient adaptability and flexibility of Foucaldian analysis ‘is to be celebrated’ (Rose, O’Malley and Valverde, 2006: 100) is self-defeating, since this pursuit of a ‘looser’ relationship to Foucault’s work, dropping in on it from time to time to borrow an insight or deploy a concept, has itself become overbearing. In many cases it has become the *only* normative principle that can be advanced by those wishing to remain within the field of Foucaldian scholarship.

The overall difficulty with this fealty to Foucault’s theoretical endeavour is that its predilection for patiently descriptive theoretical investigations incurs a retreat from clear statements about possibilities for political action, and fosters a growing detachment from any impulse for change. From a fear of bold pronouncements – as if any revealed political commitment will betray a lack of intellectual sophistication, doing violence to Foucault’s thought, constraining the supple productiveness of his ideas, translating them at the very worst into epochal and totalizing claims – theorists of this tradition learn to restrain their own impulses in what might be viewed, with some irony, as an exemplary case of self-government. More succinctly: a fear of betraying Foucault’s critical ethos has bred a fastidious and overly refined theoretical appetite.

We argue that, given our changed political climate it may be worth returning to and re-evaluating the choices Foucault once made when defining what is now a highly influential analytic frame. This article focuses on the theoretical and strategic realignment that occurred during Foucault’s lectures at the Collège de France in 1976 (Foucault, 2004[1976b]). These lectures mark the transition from his conception of power-as-war to the revised (and now dominant) framework of power-as-government. In what follows, we examine the possibility for a return to the analytic of power-as-war and argue that such a manoeuvre would restore to Foucaldian work that once productive dissonance between militancy and scholarly caution.

## Frameworks of power

To understand Foucault’s political stance, and explore possibilities for retrieving his thought’s radicalism, it is necessary to revisit the ways in which Foucault subverted conventional understandings of power, looking in particular at the options he explored, and the strategic choices he made while developing his approach. His lectures at the Collège de France in 1976 represent for us a brief but important moment, pregnant with possibilities that were left largely unexplored. During these lectures, the metaphor of war or warlike struggle as a framework for understanding power is briefly foregrounded before it retreats behind later conceptualizations. In the years that followed Foucault shifted emphasis from war to the metaphor of government. This afforded several crucial advantages in the analysis of power, and these advances are certainly ones that we would not wish to discard. In what follows we explore this transition, outline its consequences, and argue for a return to the analytic of war as a way of investing with new energy the multiple insights that have been gained through the analytic of government.

In the first lecture Foucault contrasts ‘two great systems for analysing power’, the framework of ‘contract-oppression’ and its rival, the schema of ‘war-repression’ (Foucault, 2004[1976b]: 17). The first conception of power as ‘contract-oppression’ entails questions such as: What is a legitimate exercise of power? How should we establish checks and balances to restrict power, to prevent legitimate power from degenerating and becoming oppressive? By contrast, the second conception, which seeks to analyse power in terms of *war-repression*, does not concern itself with juridical questions of legitimacy. Here, *repression* ‘is not what oppression was in relation to the contract, namely an abuse’. On the contrary, repression is simply the experience of a ‘perpetual relationship of force’ from which there is no escape into the neutral realms of legitimate power (ibid.).

Foucault locates his early work within the ‘struggle-repression schema’ (2004[1976b]: 17), though he immediately separates the two terms in this pairing. On repression Foucault provides the following clarification: in his *History of Madness*, it was sufficient to think of power as something that represses (Foucault, 2009a[1961]). Before the 19^th^ century ‘power was exercised over madness primarily in the form of exclusion’ and thus madness was a ‘privileged case’ (Foucault, 1996a[1977c]: 207). Nevertheless, we find that Foucault had ‘long been suspicious of this notion of “repression”’ (Foucault, 2004[1976b]: 17). It was through an interest in prisons and sexuality that Foucault was prompted to look beyond power-as-repression and develop an analysis of its more ‘positive’ and productive effects (Foucault, 1996a[1977c]: 207). Indeed, one could say that *Discipline and Punish*, published in 1975, marks his conceptual transition from repressive to productive power (Foucault, 1998[1976a]). During a lecture delivered one year later the productive aspects of his account of the prison are given far greater prominence (Foucault, 2009b[1976c]), and by the time of his first book on sexuality in 1976 the repressive hypothesis was under sustained attack (Foucault, 1998[1976a]).

So what about war, the other half of his war-repression schema? Here Foucault (2004[1976b]: 18) remains ambivalent in the first few lectures of 1976. In subsequent commentaries, this ambivalence on the question of war is rarely noted, or, if noted, it is assumed that this ambivalence is the product of an intellectual crisis that was later overcome (e.g. Patton, 2013). As we outline below, there seems to have been a retrospective tendency to assume that during the 1976 lectures Foucault introduced a warlike interpretation of power purely in order to reject it (i.e. by drawing attention to its dangers). Readers of Foucault-inspired research are then reminded that once Foucault returned from his sabbatical in 1978 he preferred to think of power in governmental terms. According to this view, the 1976 lectures become nothing but a point of transition from power-as-discipline to power-as-government.

The problem with this perspective is that the lectures of 1976 are, in effect, misunderstood. This reading presumes that power-as-war was not a candidate for serious conceptualization – it was just an idea, a possibility that Foucault was exploring. By contrast, we propose to draw attention to Foucault’s ambivalence at this point. While it is undoubtedly true that in subsequent lectures Foucault laid great emphasis on power-as-government, or on a governmental interpretation of power, it is wrong to assume that Foucault passed from power-as-discipline to power-as-government, without seriously entertaining an analytic framework of power-as-war. To the extent that Foucault ever rejected a warlike interpretation of power, this rejection came only later.

In the lectures Foucault poses the following questions. ‘Are we really talking about war when we analyze the workings of power? Are the notions of “tactics,” “strategy,” and “relations of force” valid?’ Should we invert that well-known statement attributed to Carl von Clausewitz (1997[1832]) where war is described as a ‘continuation of politics by other means’? Would it not be better to ask whether ‘politics is the continuation of war by other means’ (Foucault, 2004[1976b]: 15)? If some sort of ‘permanent war’ is in operation, what is the nature of this struggle that persists beneath the ‘calm order’ of our day-to-day lives (ibid.: 46–7)? Referring to ‘class struggle’ as the typical response to this last question, Foucault later declares that what he finds ‘striking’ is how Marxists tend to ‘pass over in silence what is understood by *struggle*’ (Foucault, 1980c[1977d]: 208). By contrast, these lectures would examine what he calls ‘Nietzsche’s hypothesis’, the idea that at the ‘basis of the power-relationship’ is ‘a warlike clash between forces’ (Foucault, 2004[1976b]: 16). The type of war metaphor that Foucault has in mind here is the metaphor of civil war or insurgency where direct confrontations between amassed forces are rare. Foucault viewed grandiose accounts of power – accounts influenced by the metaphor of a clearly defined battlefield of opponents – as inappropriate, proposing instead to explore struggle in all its terrestrial and contradictory detail.

Foucault’s appeal to a specific model of war in these lectures remains, for all its analytic possibilities, inconclusive. Despite his focus on the warlike skirmish in all its localized, aleatory and uncodified forms, that is, despite his appeal to a metaphor of warlike relations that must be contrasted to grander depictions of amassed forces and great repressions; what Foucault does not offer in his comparatively brief analysis is an exploration of how the analytic of war is to be decoupled from a repressive conceptualization of power in the war-repression schema. For anyone wishing to develop Foucault’s insights on war, this remains a problem to be addressed. In the first lecture, the audience is told that the ‘problem of war in civil society’ will be one that Foucault can only ‘begin’ to examine this year and may be working on for ‘years to come’ (2004[1976b]: 18). As we now know, this project was soon abandoned.

## From power-as-war to power-as-government

Three months after his last lecture in March 1976, Foucault (2002a[1977b]: 124) remarks that it is ‘astonishing to see how easily and self-evidently people talk of warlike relations of power or of class struggle without ever making it clear whether some form of war is meant, and if so what form’.^3^ By 1978, he was less equivocal, now complaining that ‘discussions on political subjects are parasitized by the model of war’ (Foucault, 2002c[1978b]: 297). One difficulty with war as a cipher for power is that it threatens to polarize discussion. We are tempted to think in terms of victors and vanquished, oppressors and oppressed, in a way that purges analysis of any subtlety and useful insight. Thus Foucault later explains that instead ‘of an essential antagonism, it would be better to speak of an “agonism”’ – ‘a relationship that is at the same time mutual incitement and struggle; less of a face-to-face confrontation that paralyses both sides than a permanent provocation’ (Foucault, 2002e[1982]: 342). Here Foucault qualifies, but nevertheless retains, a concept of warlike flux in his conception of power. It is now, though, subsumed within his notion of power as ‘a question of “government”’ (ibid.: 341).

Another problem with power-as-war can be derived from Foucault’s remarks on violence. When ‘we speak of violence, and this is what bothers me about the notion, we always have in mind a kind of connotation of physical power’ which ‘allows one to think that good power’ is power that is ‘not permeated by violence, is not physical power’ (Foucault, 2006[1974]: 14). Foucault was at pains to demonstrate that the violence of power could at the same time be rational, calculated and controlled.

It would appear to us that there are two potential options when seeking to break down the dichotomy between violent and ‘reasonable’ power. One option, following Nietzsche (1998a[1886]: I, § 2), is to explain how one thing can ‘arise from its opposite’ (truth from error, reason from violence, etc.). This is not to make an ontological claim about power or reason (e.g. ‘power and reason are essentially warlike’). The provocation – war is the origin of reason – is simply a device to help us escape equating power with the absence of reason, or reason with the absence of power. One could argue that this was Foucault’s strategy in 1976. The other option is to travel outside the dichotomy and find a third term. Taking *war* as the metaphor for violent power, and the juridical *contract* as the metaphor for ‘reasonable’ power, Foucault would, as we have seen, later opt for a third term, that of *government*, which would allow him to escape the troubling binary.

Foucault developed this governmental theme in his lectures of 1978 and 1979. By the end of the 20^th^ century this had spawned an influential and prolific body of research which we describe, for the sake of brevity, as ‘the governmentality approach’.^4^ Within this loose grouping, it is now axiomatic that ‘the relationship proper to power’ should be sought ‘not on the side of violence or struggle…but, rather, in the area of that singular mode of action, neither warlike nor juridical, that of government’ (Foucault, 2002e[1982]: 341). Playing on the double meaning in French of the verb *conduire* which means to lead or drive, and *se conduire* which means to behave or conduct oneself, it follows Foucault’s suggestion that power is all about the ‘conduct of conducts’. Power is ‘less a confrontation between two adversaries’ than a ‘question of “government”’. ‘To govern, in this sense, is to structure the possible field of action of others’ (ibid.). This conception takes us right from the macro-government of states down to the micro-government of communities, families, children, souls and so on.

In abandoning the notion of power as a kind of violence, and opting for a third term, that of government, there has been an important, and to our minds a politically detrimental, shift in emphasis. In 1976 Foucault was explicit in noting how something can arise from its opposite, how structures can be ‘built on top of this web of bodies, accidents, and passions, this seething mass which is sometimes murky and sometimes bloody’ (Foucault, 2004[1976b]: 54). Here in Foucault’s work there was perhaps a convergence between the influence of Nietzsche’s ‘how much blood and horror is at the bottom of all “good things”’ (1998b[1887]: II § 3) and Marx’s ‘capital comes dripping from head to toe, from every pore, with blood and dirt’ (1990[1890]: 926). This emphasis retains within view the *continued violence* of power even when it becomes codified in governmental devices. It is a perspective on the nature of power that perpetually incites radical critique, rousing the theorist who holds this view from intellectualized detachment. Yet today’s governmental analysts diligently avoid bold and incendiary proclamations as if reference to the violence of power would betray a lack of sophistication. With violence suppressed, the importance of *struggle* beneath systems of government is given little attention, and the possibility of an equally violent response is silenced.^5^

Mitchell Dean speaks of governmental genealogy as ‘endogenous to a political system, and practised in a time of limited political adversity’ (1999: 45). This characterization of the political context in which genealogy operates is curious, but it is an understanding that is shared by, is central to, the governmental analyses of those neo-Foucaldian researchers who, in his view, form a ‘new sub-discipline within the social sciences and humanities’ (ibid.: 2). What is curious here is the notion of ‘limited political adversity’, a condition that, it is clearly implied, obtains in advanced liberal democracies. The term may indeed be justly descriptive of the limited possibilities for active political dissent that typify such societies, the restrictions that liberal government (in its fullest sense, involving its synergetic relationship with capitalism) places on adversarial politics, but it is surely very complacent concerning the violences of this ‘reasonable’ power system. For Dean ‘limited political adversity’ is just something to be accepted and whose parameters one is obliged to work within. It is this complacency that we critique in this article and which we seek to disturb by re-evaluating the notion of power-as-war. We share Mitchell’s unease with ‘the prophetic and apocalyptic tone’ of much social and political theory but have no problem with such theory’s recognition of the persistence, in varied forms, of struggle in the management of human affairs and the violences that are enacted under the banner of liberal democracy. These are the necessary recognitions that are all too often invisible in the analyses of governmental researchers.

In tune with his subsequently less martial interpretation of power, Foucault’s earlier interest in power-as-war has received very little attention. The dominant position seems to be to accept without question the merit of Foucault’s own shift in emphasis away from power-as-war. When power-as-war is mentioned, it is swiftly rejected as an experimental dead-end that was discarded on the way to a better understanding of power, as government (Dean, 1999: 25). More detailed investigation has done little to upset this balance (see Pasquino, 1993), allowing a Foucaldian mainstream to maintain its view that power-as-war has little to offer – or that what it does offer is of a purely negative order. Within this view Foucault’s interest in power-as-war is located somewhere between an irrelevance and a potentially dangerous misjudgement.

## The 1976 lectures

Foucault’s later playing-down of the strikingly prominent war metaphor in the 1976 lectures is not the only reason why we might be encouraged to dismiss it. There is a more immediate difficulty located in the very *structure* of the 1976 lectures. Here Foucault’s treatment of the war metaphor is complicated by his overlapping methodological and historical attachments.

Literature dealing with the lectures of 1976 is relatively sparse and tends to focus on the more prominent historical narrative contained within them. See, for example, the work collected by Morton and Bygrave (2008). Marks (2000: 135) provides a rare exception when he argues that the ‘unstated dynamic’ of these lectures is their methodological content. However, Marks is quick to adopt the conventional line in dismissing Foucault’s methodological interest in power-as-war as a mistake that is beyond redemption. For us, these methodological attachments are, roughly speaking, genealogical in the sense outlined by Ansgar Allen (2014). They have, nevertheless, been obscured by the more obvious historical narrative of the lectures. In summary, these two positions are as follows:

1. Methodology: these lectures rework the concept of power by addressing the ‘war’ element of the war-repression schema. This level of interest is broadly speaking *methodological* in that Foucault sets out to investigate how a range of marginalized historical figures conceptualized power-as-war and investigated its effects.
2. Historical narrative: in these lectures Foucault provides a tentative and experimental history of historiography. He recalls a time when notions of war and struggle were used to conceptualize the past and contest the present. He goes on to explain how in the 19^th^ century this discourse was eventually coopted and internalized by the modern state. By implication, it would seem that to speak in terms of war today is to adopt an outmoded discourse. This discourse has been assimilated and thus no longer provides the resources upon which we can construct a critique of contemporary power. Such, indeed, is the conclusion we arrive at if the second *historical* argument is taken as an epochal claim, and thereby allowed to eclipse the first *methodological* one. We will explore this process of eclipse in more detail below.

We recognize that the division we effect between the methodological and historical levels places us in danger of producing a simplifying reduction of Foucault’s argument, or it would if we were proposing that it revealed a damaging and invalidating split within his thought. We have no such intentions. Indeed, we accept that Foucault’s analytic framework often reflected his historical focus, which is why, for example, Foucault adjusted his analytic techniques away from a conception based on power as a repressive force (from power as something that is possessed by and concentrated in powerful individuals) when examining an historical period in which other, less centralized modes of power were becoming prominent. Foucault (2007a[1978d]: 107) was though perfectly explicit in stating that sovereign power was not replaced by new techniques; it was reinvested alongside new developments in governmental management. Perhaps surprisingly, Mitchell Dean (2007) has worked to rehabilitate the analysis of sovereignty in governmental theory. We argue that there is a similar potential to rehabilitate a warlike conception of power, which does not automatically become redundant in an era that is perceived as increasingly ‘governmental’. The difficulty, for us, is that Foucault’s *historical* analysis in the 1976 lecture series has overshadowed and thereby diminished the significance, the usefulness, of his *methodological* innovation in perceiving power in warlike terms. And yet the neglected methodological focus of these lectures has, we argue, the potential to restore to Foucaldian critique what it has since lost, this being an uncomfortable but productive relationship with militancy.

## Foucault’s history of historiography

In the development of Foucault’s *historical* narrative we are presented with a tentative study of the historian. Foucault identifies three phases in the writing of history. His study begins by exploring historical discourse in its ceremonial function, where history was used to justify and reinforce existing power. History would show how existing sovereigns were preordained with an indisputable right to rule by ‘extracting from’ all the ‘vicious and violent accidents [of the past] which are linked to error, a basic and permanent rationality which is, by its very essence, bound up with fairness and the good’ (Foucault, 2004[1976b]: 55).

At various points in and around the 17^th^ century another mode of retelling the past began to emerge in the margins, where it remained until finally disappearing in the 19^th^ century when it would be ‘denounced as the discourse of a biased and naive historian, a bitter politician, a dispossessed aristocracy, or as an uncouth discourse that puts forward inarticulate demands’ (Foucault, 2004[1976b]: 58). This was a form of ‘counter-history’, where the implicit assumption that people and sovereign were one was dropped as histories of races began; ‘race’ is used here in its original French sense, meaning: ‘membership of the royal (and noble) families of the Middle Ages’.^6^

Within these rival historical narratives, the role of sovereign history was inverted and fragmented. It was inverted because the vicious and violent accidents of the past were no longer linked to error; they were no longer explained away so as to reassert sovereign right. It was fragmented because history was now providing the source material for rival and contending accounts. These accounts were ‘deployed within a history that has no boundaries, no end, and no limits’. The ‘drabness’ of histories that were organized around ‘a few basic, stable principles’ was replaced by something more fiery and ephemeral (Foucault, 2004[1976b]: 55). Multiple histories were now told in support of rival factions that were laying claims to their respective legitimacy and importance. These rival accounts might argue on behalf of a hitherto neglected but noble lineage, or they might tell a more discontinuous history that accounted for their current subjection. There was no longer one history for all; rather, history became an oppositional tool by which the status quo could be challenged. As forgotten struggles were unearthed, and as the ‘blood that has dried on the codes’ was rediscovered (ibid.: 56), these multiplying histories of races broke apart from the old homogenizing history of the sovereign.

Through a detailed study of historians such as Henri de Boulainvilliers, Foucault was able to show that these histories were not always simple tales of ‘victors and vanquished’. At times they were far more finely drawn, analysing fluctuations of power, showing how sources of strength could become principles of weakness. They were able to explain how ‘a certain relationship of force’ was slowly ‘and for obscure reasons, inverted’. This led to ‘the emergence of a diversity of struggles’ and ‘shifting front lines’ as the analysis proceeded towards finer historical distinctions. Larger struggles were ‘divided and transformed by multiple channels’. The relationship of war became ‘part of every social relationship’ fragmenting into ‘thousands of different channels’, revealing war to be ‘a sort of permanent state that exists between groups’. History now appeared as a ‘calculation of forces’ (Foucault, 2004[1976b]: 160–2), and historical discourse became a political device, having established in effect a form of ‘historico-political continuum’ where the recounting of history was ‘not simply a matter of describing a relationship of force’ – it was an attempt to adjust ‘current relations of force’ by interrupting present politics with revised historical understandings. History thus became ‘a knowledge of struggles’ that was deployed ‘within a field of struggles’. There was now ‘a link between the political fight and historical knowledge’ (ibid.: 171).

It is precisely this connection, where the discourse of historical struggle became tied to a political fight, that Foucault was, in his own words, ‘praising’ (2004[1976b]: 65). Here Foucault expresses a *methodological* commitment: it is the *function* of history as an oppositional tool that he admires despite such discourse being used as a tool of rival nobilities, even though, as Foucault observes, it was often ‘basic, clumsy, and overloaded’, and contained ‘the most insane hopes’ (ibid.: 56–7). There is a certain attraction to the bold cut and thrust of these counter-histories and the reader of these lectures may well be disappointed to discover that during the 19^th^ century, the counter-history Foucault celebrates was transformed into something rather different, and this is where any praise of his comes to an abrupt halt. This discourse was coopted and transformed in two important respects:

1. According to Foucault’s tentative genealogy, in the 19^th^ century the multiplex discourse of struggle was reduced to the revolutionary idea that one final battle could end all struggles and result in utopia. This, in effect, inaugurated the revolutionary modernism that Foucault would expend such great effort problematizing.^7^
2. During a similar period rather than being recoded in class terms, as Marxist analysis might have wanted, counter-history was recast as attempts were made to ‘recode the old counter-history…in terms of races – races in the biological and medical sense of the term’. This ‘biologico-medical perspective’ crushed ‘the historical dimension that was present’ in the old discourse (2004[1976b]: 80). Coupled to the idea of biological purity, this coopted discourse led to the late 19^th^-century rise in state racism (including, eventually, Soviet state racism^8^). Struggle was recast in terms of modern racist discourse, which redefines social groups in terms of the biological threat posed by them to overall biological purity and health. As a result, the older and distinctly pre-biological discourse of race war once employed by counter-history was conceptually reconfigured as an ‘internal war that defends society against threats born of and in its own body’ (ibid.: 216). In this switch from an ‘emancipatory project to a concern with purity’, struggle transformed into a fight for the overall well-being of a state, a biopolitical fight to preserve the state from impurity or contamination. Counter-history, a political tool of rival and contending groups, was thereby turned back on the unruly and mutinous dispositions of those who had once forged it. It was reduced to a tool of state, a rationale for removing ‘heterogeneous elements’ (ibid.: 81).

So, to bring this to a conclusion, as one commentator and editor of Foucault observes: ‘the lectures not only provided the promised historical celebration of militant thought but also exposed the limitations and immense dangers of that style of thought’ (Gordon, 2002: xxii). According to Patton (2013), this lecture series was intended by Foucault as a response to those on the left who were now taking up arms (e.g. the assassinations and kidnappings carried out by groups such as the Red Brigades and the Red Army Faction). Hence Foucault’s genealogy of historical discourse, in which struggle is either reduced to revolutionary violence or internalized within discourses of state, is used to cast doubt on power-as-war as a *methodological* attachment. It would seem, from this perspective, as if the militancy of the counter-historical perspective is not to be trusted, since it is vulnerable to cooption and likely to lead to an escalation in violence. For us, while these observations are important, they still do *not* imply an absolute rejection of power-as-war. Instead they invite us to investigate more precisely what we mean when we assert the necessary violence of power. We are also invited to investigate the strategic consequences of taking such a position. For example, the following questions become pertinent: (1) to what extent must a form of power analysis that models itself on disparate struggles, inevitably find itself becoming reduced to a potentially tyrannical singularity; and (2) to what extent might genealogical work which abjures militancy find itself becoming internalized within the systems and operations of control that flow through the social and which the state attempts to coordinate?

These are both considerations that we should be aware of, serving to warn us that a form of power analysis which bases itself within a framework of power-as-war, can be coopted by repressive forces. While the lectures of 1976 do not constitute a rejection of power-as-war, in the years that followed there was certainly a subtle shift in Foucault’s approach to power. This shift has nevertheless been magnified and distorted by those who insist on a rejection of the war metaphor. We argue that through a close reading of the lectures it becomes less obvious that we should reject the framework of power-as-war out of hand.

## On ‘not being governed so much’

It is, in our view, regrettable that Foucault’s sensitivity to the complacent assumptions of militant discourse has been translated, in the work of his followers, into a scholarly effort to purge critique of almost all normativity, as if any goal or direction, any normative commitment, however mild, would perpetuate a dangerous naivety. Here the term ‘normativity’ stands for any value-based commitment that has the pretence to universality.

While many normative commitments may appear trifling at first sight, those who adopt a strict Foucaldian line will remind us that normativities, however minor, often presume access to a trans-historical, evaluative position or figure (such as the figure of ‘man’ – see Foucault, 1985[1966]). This presumption affords those claiming access to this evaluative standpoint the unique but nevertheless illusory privilege of standing outside time and present context in order to pronounce a verdict upon the conditions of the present. Norms are also, following Foucault’s argument in *Discipline and Punish*, underwritten by disciplinary procedures that silently secure their acceptance (Foucault, 1991[1975a]). In a biopolitical twist, norms are even now derived from the societies in which they are said to occur, helping naturalize normativities and hence raise them above critique (Foucault, 2007a[1978d]). Normativities are insidious to the Foucaldian, often subtle in their operation but monstrous in their effects.

To adopt a normative commitment, so the argument goes, is to ‘subordinate oneself in the name of an external code, truth, authority or goal’ (Rose, 1999b: 283); it is to become unwittingly ‘hard-wired’ to a political perspective (Rose, O’Malley and Valverde, 2006: 101). According to those who subscribe to this (dominant) form of post-Foucaldianism, our normative commitments are always born of and limited by our present. The oft-repeated purpose of Foucaldian analysis goes something like this: it is to expose and thereby destabilize the conceptual limits that define us in order that we may move beyond them. According to this view, when normative conventions form the basis of critique a species of norm-induced blindness arrives, insulating us from the sort of radical decentring that is required if we are to fundamentally change the present. Indeed, the notion of ‘critique’ itself has been rejected by members of this school of thought. We are told that the Foucaldian genealogical approach offers ‘not critique’ but studies that are ‘critical’ in a different sense, in that they ‘identify and describe differences and hence…help to make criticism possible’ (ibid.). Though we share Foucault’s suspicion of totalizing worldviews and their ‘claim to escape from the system of contemporary reality so as to produce the overall programmes of another society’ (Foucault, 2000b[1984a]: 316), this methodological injunction to avoid normativities carries with it an oddly normative note of interdiction.

As is well known, the debate concerning normativity attempts to adjudicate between a persistent critique of Foucault, made most tellingly by Jürgen Habermas, and those who claim that Foucault has been misunderstood.^9^ Habermas argued that without a normative foundation, Foucaldian critique is unable to answer the question ‘*Why fight?*’ Without a positive vision to work towards, it is unable to suggest why we should ‘muster any resistance at all’ (Habermas, 1994[1987]: 95). Why not simply adapt to power instead?

While we believe that the case is far from closed, Habermas’s critique misfires to the extent that it is concerned with separating legitimate from illegitimate power so that one might pursue the former and resist the latter. Foucault rejected this entire conceptual framework as wedded to a contractual conception of power. This conception – ‘articulated around power [viewed] as a primal right that is surrendered, and which constitutes sovereignty’ – elevates the social contract as the ‘matrix of political power’ (Foucault, 2004[1976b]: 16–17). The contractual conception of power serves as a regulating idea, constraining discussions concerning power into normative questions of right, responsibility and obligation. This conception of power is, from a Foucaldian point of view, unable to account for the multiple, entangled forms in which power manifests itself. More importantly, by escaping the implicit normativities of contractual power, Foucault was able to gesture to more radical forms of political engagement. Take, for example, these remarks from an interview in 1971:

[W]e can’t defeat the system through isolated actions; we must engage it on all fronts – the university, the prisons, and the domain of psychiatry – one after another since our forces are not strong enough for a simultaneous attack. We strike and knock against the most solid obstacles; the system cracks at another point; we persist. It seems that we’re winning, but then the institution is rebuilt; we must start again. It is a long struggle; it is repetitive and seemingly incoherent. But the system it opposes, as well as the power exercised through the system, supplies its unity. (Foucault, 1980b[1971b]: 230)

Evident here is an acknowledgement of the need for multiple struggles that together constitute a radically fragmented form of collective action. Here, in this quote, we find a sober recognition of the necessarily extended nature of this political fight, which must take account of the multiform and protean nature of power. It is precisely this militant aspect that remains neglected in neo-Foucaldian work, where his accompanying critique of utopianism several lines later in the same interview (‘to imagine another system is to extend our participation in the present system’), is more regularly cited (1980b[1971b]: 230).

While it is axiomatic among the defenders of Foucault to reject restrictive conceptions of power and guiding visions of the future as destructive to the project of Foucaldian critique, some writers have, nevertheless, hinted at a political orientation. Here we return to the 1990s, for this was the decade that secured Foucault’s reception in the English-speaking world. As Rose, O’Malley and Valverde (2006: 89) argue, this was the period in which the dominant ‘analytical framework of governmentality…assumed the form’ that ‘it takes today’. Our claim is that the implicit politics we identify with this ‘analytical framework’ still dominates the field today: according to David Owen (1995: 501), the ‘architectonic practical interest of genealogy’ is autonomy. Thomas Osborne (1999: 47) speaks of ‘self-creation’, while Nikolas Rose, following his analysis of the ‘death of the social’ and apparent denial therefore of any politics that might be built from this (now absent) well-spring (Rose, 1996), suggests that in this post-social age ‘each person’s life should be its own telos’. This would be the ‘minimal normativity’ to which he could subscribe (Rose, 1999b: 283). From this governmental perspective some ‘ways of governing are intolerable precisely because they exclude the possibility’ of self-formation (ibid.).

The apparent individualism of these statements, which seem to indicate the persistence of a liberal humanist hankering after autonomous, free selfhood, suggests that these writers are unable to achieve sufficient critical distance from present-day political discourse. Even within the governmentality school this has caused concern. Mitchell Dean (2007: 86) has criticized some writers for appearing to ‘endorse’ liberalism as ‘relatively benign’, claiming that there is in their work a ‘narrowing of the relationship’ between a liberal approach to government and ‘the analysis offered by governmentality studies’. Seen perhaps as somewhat of an outlier, Kevin Stenson (1998) spots the potential for an identification of the governmentalist project with liberal values, arguing that governmentality studies be deployed as a deliberate tool to reinforce liberalism and promote autonomy: as defenders of ‘the most civilized tolerant framework of living yet devised’, members of this school should also set themselves against ‘radical critics’ who ‘may at times pose a threat to liberal values and principles’ (ibid.: 350).

Whether Stenson embodies the underlying truth of the governmental position in the sense that his thinking performs a distillation to the fundamental assumptions of neo-Foucaldian critique, or represents a deviation from the vocation as suggested by Dean, there is likely to be a basic difficulty with this school of thought. The Foucaldian version of the subject, as elaborated in *The Archaeology of Knowledge* (Foucault, 2002f[1969b]), is ‘a radically decentred subject whose conditions of existence are relational, dispersed, never finally given’ (Goddard, 2010: 351). There are telling affinities here with the liberal/neo-liberal subject, the individual best fitted for performance under the conditions of an advanced global capitalism, a subject that is endlessly adaptable, flexible and rootless. Small wonder, perhaps, that neo-Foucaldians find it difficult to put a space between themselves and the neo-liberal scheme of things. This affinity all too often limits neo-Foucaldian research to descriptive analysis and renders it incapable, it seems, of anything like a vigorous political critique.

Perhaps all this should come as no surprise. After all, what sort of resistance does power-as-government imply? In 1978 following his switch to the analysis of power-as-government, Foucault offered a succinct definition: alongside a ‘multiplication of all the arts of governing’ there has developed ‘the art of not being governed so much’. This, we are told, is the ‘preliminary definition of critique’ (Foucault, 1996b[1978c]: 384). It is a definition of critique that is notably close to the liberal order of discourse, which is preoccupied with the problem of too much government, of inserting economy into political practice (Foucault, 2008[1979b]). Indeed, Foucault is quite clear that this critical attitude, the art of not being governed so much, ‘is at once partner and adversary of the arts of government’. This critical attitude is both a means of challenging the arts of government, and a way of refining them by ‘finding their right measure’ (Foucault, 1996b[1978c]: 384).

The governmentalist conception of critique is, therefore, ambiguous in its very constitution, as Foucault makes abundantly clear. This ambiguity continues to be expressed in the work of his followers. In his exposition of the ethics and politics of the later Foucault, Osborne (1999: 53) puts it like this: to the extent that Foucault had a political orientation, it was based ‘not on our status as human beings but on our status as governed beings’. While ‘we may not share an essence, a soul, an identity or any other fixed attributes with others’, Rose adds, ‘there is one status that we do share, and that is our status as subjects of government’ (Rose, 1999b: 284). Rose declares that we even ‘have the right, as governed subjects’ to contest the practices that govern us in the name of freedom (ibid.: 59–60). The idea of a ‘right’ in this context has a specific meaning and does not necessarily imply an essential humanism, according to which we are somehow born with inalienable rights. It is only the contingent fact that we are currently ‘subject to government’ that gives us the right to collectively ‘resist government’ (Osborne, 1999: 54). Foucault puts this a little more strongly than his followers when he states that we are ‘obliged to show mutual solidarity’ as ‘members of the community of the governed’ (Foucault, 2002g[1984b]: 474–5).

For us, the difficulty with this formulation of the critical enterprise is that it is promoted by a neo-Foucaldian tradition of research that also employs a highly diffuse and pluralistic conception of government. If anything defines the governmentality school, it is the claim that neo-liberal times are not available to representation as a monolithic regime of power. The wish not to be governed ‘so much’ is thus provided with few resources for refusal from within this conceptual frame. The risk here is that the current order of things finds its fragmented reflection in a myriad of minor personal rebellions, reifying the individualizing absorption of each and all within his or her own fashioning of self. Such a conceptualization of the critical enterprise would sustain the operations of what post-Foucaldian governmentalism declines to recognize as a very real and dangerous regime of power, the neo-liberal doctrine that licenses the actions of globalized capitalism. Of course, this is also a regime that is more varied in its forms, its instrumentalities and its coordinating impulses than a global critique can comprehend.

We would argue that the analytic perspective afforded by a concept of power-as-war entails a practice of critique that has far greater potential for challenging existing dispensations of power in general, and, in particular, for interrupting liberal complacencies, than the generally acquiescent formula described above. The real advantage of power-as-war is that it emphasizes the basic Foucaldian point, which is that when peace arrives, so does domination. If a Foucaldian critique of power has any normative content worth pursuing, this should be located in:

1. an overall suspicion of peaceful appearances: where benign peace is just a cover for ‘blood that has dried on the codes’ (Foucault, 2004[1976b): 56) and despotic peace is the same wound but without the bandages;2 a qualified promotion of militancy: rejecting militancy that either embodies a tendency to peace or a tendency to domination.

This position need not contradict the governmental wish to be governed less. However, it does imply an important switch in emphasis, from individual subjective resistance to collective material refusal, from a personal desire to take control over the practices that form the self, to a communal project that seeks to promote disorder and introduce greater tactical polyvalence to those practices of self-production.

## From ‘*Why fight?*’ to ‘*How fight?*’

If one knows how to move, the absence of a schema is not an obstacle but an opportunity. (The Invisible Committee, 2009: 19)

It might be productive to begin with the strategic question ‘*How fight?*’ That is, what would be the conditions of conflict most suited to Foucaldian critique? This would avoid what Foucault referred to as Enlightenment blackmail, a form of conceptual trickery that imposes the constraints of ‘a simplistic and authoritarian alternative’ (Foucault, 2000b[1984a]: 313). To begin by asking whether a historically contingent, embattled and anti-foundational form of critique can ever answer Habermas’s normative ultimatum, ‘*Why fight?*’, is to set out in the wrong direction. It presumes access to a trans-historical, evaluative position or figure. Foucault’s reply is that we should become ‘less concerned with *why* this or that than with *how* to proceed’ (Foucault, 2007b[1972b]: xiv).

Described as ‘the first key move’ of a Foucaldian analysis of power, this switch ‘from why to how’ (Miller and Rose, 2008: 8) has been applied elsewhere to effect a ‘breach of self-evidence…making visible a singularity at places where there is a temptation to invoke a historical constant’. To ask *why* things occur entails questions such as: Who invented this technique? What wider social process does it represent (capitalism, social disintegration, modernization, class conflict, etc.)? Whose interests does it favour? And so on. By contrast, Foucault (2002b[1978a]: 226–7) adopts a resolutely descriptive tone in explicating the ‘how’ of power, seeking a ‘multiplication or pluralization of causes’.

Applying this logic to its own enterprise, we might argue that neo-Foucaldian scholarship should not begin by telling us *why* it generates a particular form of critique (i.e. whether it is for emancipation, human rights, equality, autonomy, freedom, etc.). Instead, it could investigate *how* we could better practise critique, what are the conditions conducive to the critical enterprise. The issue of normativity does not vanish, but it is deferred. It is no longer assumed that *why* comes first, or that to answer why provides the necessary foundation upon which all else must rest. The challenge now, is to develop new normative commitments *through collective struggle and deliberation.* It is to operate without the restraints and rationales imposed by a pre-existing normative political programme, which will always presume too much, sharing in its formation many aspects of the regime to be overthrown.

So how does a Foucaldian critic fight? In what has become a powerfully influential mode of engagement in Foucaldian scholarship, the Foucaldian critic barely fights at all. The typical neo-Foucaldian scholar ordinarily remains distant from struggle, urging caution, intellectual modesty and analytic reserve.

There are, nevertheless, occasional gestures in the governmental corpus towards a collective project, a potentially unifying Foucaldian praxis, but these gestures are rare and often highly tentative. For example, towards the end of his paper on Foucault’s critical ethos, Lemke (2011) situates Foucault’s enterprise within the context of a common ‘normative horizon’, which, by implication must make the activity of critique a common endeavour. Even though Foucault’s critical ethos will continue to operate without a ‘substantive idea of the “good life”’, our shared existential horizon means that such anti-foundational critique is never, purely, ‘an individual option’ (ibid.: 38). The weakness of this position, for us, is that it remains subservient to a social and political horizon that defines where and when political issues become collective concerns. While Lemke, in a penultimate paragraph, hints at a role for ‘passion and desire’ in driving critique, the precise role of passion, and its relation to critique, remain unexamined (ibid.: 40).

Despite occasional moments such as these, the overriding tendency has been to ‘point to a certain modesty in Foucault’s conception of political activism’ (Osborne, 1999: 55). This modest attitude rejects, as Osborne argues, ‘a need much trumpeted today – namely, for a new “big idea” to help us into the next millennium’ (ibid.: 56) and recommends instead a more inventive ‘stylization of oneself – as an individual or as a collectivity – in relation to government’ (ibid.: 54). Again, these are passing remarks without any connection to political praxis. They are also limited, once again to a critical position that is subservient to a horizon defined by government.

It is remarkable that even the foremost proponents of what is for us a tamed critical ethos, the ‘governmentality’ theorists in Britain and elsewhere, have been subjected to their own methodological critique. They have been criticized for failures to exercise sufficient Foucaldian caution in their research. It is clear that within this intellectual landscape, even the most devoutly cautious intellectual practitioners are not immune from attack. Stephen Collier (2009) argues that governmental theorists are, despite their methodological intentions, still prone to global pronouncements. A yet more ‘supple analysis’ is possible and necessary, one that shrinks still further from a ‘diagnostic style’ of thought given to ‘epochal announcements and totalizing claims’ (ibid.: 79–80). Foucault himself, Collier claims, was prone to these attacks in his earlier work, and yet managed to restrain this impulse in his last years through a diligent investigation of ‘patterns of correlation in which heterogeneous elements – techniques, material forms, institutional structures and technologies of power – are configured’ (ibid.: 80). This was the work of a more restrained Foucault, so the argument goes, who explored assemblages of power and ‘broad configurational principles’ (ibid.). This was a Foucault who had become resolutely descriptive, who had become analytically cool, no longer relying on implicit mythologies of oppression, nor appealing any more to a broader politics of refusal.

A typical response to Collier would be to assert that Foucault was never given to epochal claims, or if he was, that these were minor slips in an abiding project of carefully measured statements. Our position is somewhat different, as we sense a more consistent and enduring tendency in Foucault to combine meticulous investigation with the urgency born of a militant impulse that was never entirely disavowed. Indeed this productive dissonance is, for us, what makes his work so engaging and provocative.

Unlike Collier we would prefer to give the most eminent scholars of the ‘governmentality’ school their due as having in many cases successfully achieved the critical distance they seek. It is only regrettable that the critical enterprise they promote is the remainder of a more vigorous, once bolder form of engagement. In the hands of its originating thinker and for a brief period during the early 1970s, this critical work formed a provocative and stimulating countervailing force against the tendencies towards revolutionary singularity, reductive theoretical unity, and transcendence found in the doctrines and politics of Marxism, Maoism and other radical left movements of the post-’68 era. Foucault nevertheless felt that he had been too easily adopted by its self-styled radicals, and so towards the end of that decade, there was a lexical and analytic switch from the more openly militant terminology of ‘power, war and discipline’, to the more ambivalent terminology of ‘governmentality, security and biopower’. Those working with Foucault in the late 1970s at the Collège de France welcomed this transition to what they saw as a more intellectually sophisticated, less openly militant analytics of power. Indeed, co-researchers Daniel Defert, François Ewald, Jacques Donzelot and Catherine Mével were so comfortably embedded within this analytic frame that they were content to write genealogies of risk and insurance for the French Labour Ministry as the decade drew to a close. In the late 1970s Foucault’s new analytics of power had the unexpected effect of playing ‘an instrumental role in reconciling at least some members of the ’68 generation with contemporary society’ (Behrent, 2010: 617). The Nietzschean impulse that first animated genealogy had been abandoned.

We argue that, decades later, in our radically different political context any critical endeavour that relies heavily on Foucault’s analytics of power should carefully re-examine its political strategy, or risk becoming ‘at its last gasp’ mere scholarship of a variety once castigated by Nietzsche (1998a[1886]: VI § 204). If organized militancy oriented towards a singular and despotic truth were to emerge once more, perhaps neo-Foucaldian critique, as a research project practised by a militant scholarly elite, might be appropriate again. An academic cadre could once more produce monographs that work to destroy or at least cast doubt on the simplistic tales of oppression and domination foisted on us by revolutionary teleologies. But in the current climate this model of critical intervention is unable to function to any perceivable effect. It has lost the foe against which it defines itself, the unifying militant project, which has disintegrated into ‘discontinuous, particular and local critiques’ (Foucault, 2004[1976b]: 6). Neo-Foucaldian scholarship needs to adjust because, as its considerable outputs demonstrate, there is a far more dangerous phenomenon to be opposed than an anachronistic and superannuated leftist determinism – what Foucault might have termed a daemonic conjoining of instrumental rationalities and a reckless global capitalism that brings with it its own governmentalizing procedures and intentions.

Another kind of militant genealogy is required, one that has been updated to match the needs of the present. Its militancy cannot be prescribed in advance; rather it must be invented through an engagement with the process of struggle itself. Foucault was quite explicit on this point, arguing that not only would ‘political analysis and critique…have to be invented’ (Foucault, 1996a[1977c]: 211), but also this would be a ‘permanent political task’ (Foucault, 1983a: 223). His analytical and conceptual innovations produced a line of supple, sensitive enquiry, which afforded a more detailed and rigorous examination and account of governmental power than anything hitherto achieved by radical critique. This is a line of analysis along which governmentality studies continue to work. However, Foucault adds that the creation of new forms of political analysis and critique must be accompanied by the invention of ‘strategies that will allow both modifying these relations of force and coordinating them in such a way that this modification will be possible and register in reality’ (Foucault, 1996a[1977c]: 211). This call for ‘new forms of politicization’ (ibid.) remains open and demands equal attention alongside the accompanying demand for new forms of analysis and critique. Here we should avoid the temptation to adopt a division between political analysis and critique on the one hand and political action on the other. This is a typical neo-Foucaldian tendency arising from excessive fidelity to Foucault’s well-known rejection of the ‘imperative discourse’ of universal intellectuals which ‘consists in saying “Strike against this and do so in this way”’ (Foucault, 2007a[1978d]: 3). Foucault’s point here was that ‘the dimension of what is to be done can only appear within…a field of forces that cannot be created by a speaking subject alone and on the basis of his words’ (ibid.). All this may be admitted, but it does not mean that critical scholarship should confine itself to descriptive analysis and oblique political observation.

Perhaps one of the most promising departures from the governmental preoccupation with investigating the ‘conduct of conduct’ is the recent focus on the accompanying importance of what Foucault later called ‘counter-conduct’ (Davidson, 2011). Read through the lens of Foucault’s last lecture series at the Collège de France (Foucault, 2011[1984c]), counter-conduct is understood as a wilfully eccentric stylization of one’s life that plays with and subverts dominant norms. To some extent for the later Foucault, counter-conduct is to government, what resistance was to power. This lexical switch is nevertheless significant insofar as it draws attention to the specifically disruptive ethos of embodied and perverse activities. This is a welcome addition to the dissident repertoire, and yet in a liberal regime such perversions are often too easily absorbed. The obvious danger with counter-cultural styles of life is that these modes of life always risk becoming accepted by a liberal order of discourse that prides itself on its apparent permissiveness. By contrast, a wilfully militant genealogy would ensure that insubordinate activities remain unacceptable to existing dispensations of power. This genealogy would pay close attention to existing struggles in an effort to broaden their scope, enhance their sensitivity to operations of power and draw attention to attempts at cooption.

A wilfully militant genealogy would certainly cast off the intellectual restraints of prominent neo-Foucaldians who cloak their enquiries in a determined penitence for the grand and global claims of an erstwhile radicalism. As we argue above, one way of understanding this scholarly tradition is to view it as a reactive formation to the bombastic grandiosity of lockstep Marxism, in a former time the passionate attachment of many of the post-Foucaldians to whom we refer (see Frankel, 1997).^10^ Our feeling is that such persistent self-chastisement and self-limitation have involved living according to a rule that has blunted consideration of political possibility. Here a return to power-as-war would reinvigorate analysis and give it greater purchase on political actualities. As a conceptual frame it calls attention to the quality of struggle. In our current climate, this entails working towards the construction of new forms of refusal and subversion after a long period of left militant decline and strategic realignment. This would involve an investigation of the material conditions that are required to support, maintain and assist militant work, an investigation that would include genealogies of previous struggles, looking in particular at those that are relatively unfettered by theoretical globalisms, and are locally situated and responsive to the immediate logic of events.

In calling attention to the quality of struggle we are not suggesting that struggle or disorder should be elevated for its own sake as an ontological principle and goal. Rather, the task for genealogy today is to investigate the conditions that are required to enhance the potential for a more incisive and practically influential critique of a Foucaldian kind. That is, a model of political intervention that is careful to avoid simplistic, undiscriminating (or wholesale) condemnations of power, a type of oppositional practice that is open to counter-intuitive and anti-foundational critique, a militancy that attends to the multiple, diffuse and unexpected ways in which power conditions our daily lives and forms us as myopic, docile subjects, wedded in the very formation of our character to the systems that oppress us. This would be a kind of militancy that no longer has the convenience of a well-defined foe. It would be a militancy that has come to terms with the basic Foucaldian insight that there is no single agent or agentic force that is to blame, that there is no one motor of history, no unique engine of human suffering, no single exploiter or antagonist against which militancy must mobilize itself. Militancy must come to terms with all that, and yet remain mobilized, convinced as it is of the continued existence of suffering and exploitation, and of the necessity of fighting against it.

Through its opposition to the teleological schemes of 20^th^-century emancipatory thought and activism, Foucaldian critique once attempted to provide revolutionary discourse with those perspectives and tools that would liberate it from theoretical globalism’s. Today, by contrast, it must establish a different and closer relation to militancy. The *disorganization* of militant work, which has become both a fact of struggle and at times a normative ideal, demands an adjusted form of political enquiry from the left.

The distinctive contribution of work that takes its inspiration from Foucault should be to commit its subtle and nuanced armoury to this effort. The Foucaldian challenge, as we perceive it, is to augment governmental analysis with the insights and provocations of a war analytic, translated into (1) a suspicion of peaceful appearances and (2) a qualified promotion of militancy. The academic monographs of the post-Foucaldian academic still deserve careful reading, but the restrained critical ethos they have come to represent should be cast aside.

## Notes

1. Even here, liberal-democratic sensibilities can be upset, for example, by the claim that several months after the fall of President Hosni Mubarak in February 2011, protesters were dying in Tahrir Square because the original revolution was not violent enough. In other words, ‘revolution must strike [at least] twice’ (Žižek, 2011: 7).

2. ‘I often quote concepts, texts and phrases from Marx, but without feeling obliged to add the authenticating label of a footnote.’ ‘Nowadays I prefer to remain silent about Nietzsche’ (Foucault, 1980d[1975b]: 52–3).

3. This interview was conducted in June 1976.

4. See, for example: Burchell, Gordon and Miller (1991), Barry, Osborne and Rose (1996), Miller and Rose (2008) and Dean (1999).

5. Exceptions can be found outside this paradigm. See, for example, Thiele (1990).

6. See Macey (2008: 129). ‘It was not exactly racist discourse whose history I was tracing and that I was praising: it was the discourse of race war or race struggle’ (Foucault, 2004[1976b]: 65).

7. For more detail see Foucault (2004[1976b]: 58–9, 60, 78–9, 88 and 111).

8. For more detail see ibid. (83, 261–3; 1980c[1977d]: 223–5).

9. See, for example, Kelly (1994) and Ashenden and Owen (1999). More recently, efforts have been made to search for common ground between Habermas and Foucault, though it is recognized that such an approach involves recasting the views of both (Amy Allen, 2009).

10. For a retrospective account of how these neo-Marxist tendencies were overcome by those who later became ‘Foucaldians’, see, for example, Rose, O’Malley and Valverde, (2006).
